# Adiponectin receptor 1 resists the decline of serum osteocalcin and GPRC6A expression in ovariectomized mice

**DOI:** 10.1371/journal.pone.0189063

**Published:** 2017-12-01

**Authors:** Yuan-Yu Lin, Ching-Yi Chen, Shih-Torng Ding

**Affiliations:** 1 Department of Animal Science and Biotechnology, Tunghai University, Taichung, Taiwan; 2 Department of Animal Science and Technology, National Taiwan University, Taipei, Taiwan; 3 Center for Biotechnology, National Taiwan University, Taipei, Taiwan; Oklahoma State University, UNITED STATES

## Abstract

Hormonal changes that cause metabolic complications are a common problem in postmenopausal women. Adiponectin and osteocalcin are cytokines associated with glucose regulatory and insulin sensitized function in postmenopausal stages. The current study investigated the role of adiponectin signaling and osteocalcin mediated function in glucose metabolism in ovariectomized mice. In a mouse menopausal-related metabolic disorder model, overexpression of adiponectin receptor 1 improved glucose tolerance and caused resistance to body weight increase and decline of serum osteocalcin. Furthermore, adiponectin receptor 1 transgenic ovariectomized mice had higher GPRC6A (the putative osteocalcin receptor) expression in muscle tissue. Immunofluorescence indicated that GPRC6A and adiponectin receptor 1 were co-localized in mouse muscle tissues. The present finding suggested adiponectin receptor 1 can mediate the improvement of glucose metabolism by osteocalcin in ovariectomized mice. Our findings imply the possibility to ameliorate menopause-induced metabolic disorder by GPRC6A and adiponectin signaling.

## Introduction

The decrease of ovarian hormone secretion is a dramatic change in a women’s life at menopause and has been associated with metabolic complications including obesity, insulin resistance, cardiovascular disease and osteoporosis [1,2]. Several reports suggest that adiponectin (ApN) and osteocalcin (Ocn) may play a critical role in menopause [3,4,5].

ApN and Ocn are cytokines secreted from adipocytes and osteoblasts, respectively, and have been demonstrated to regulate glucose homeostasis and increase insulin sensitivity in mice [6,7]. ApN circulates in multiple forms and exerts pleiotropic metabolic functions through its receptors, adiponectin receptor 1 and 2 (AdipoR1 and AdipoR2) [6,8]. Stimulation of ApN signaling ameliorates obesity-related disease, such as type 2 diabetes, cardiovascular disease, fatty liver and osteoporosis [6,9,10,11]. Ocn enhances glucose metabolism and insulin secretion and β-cell proliferation [7,12,13,14]. Moreover, Ocn exerts its insulin sensitization in peripheral tissue purportedly through the GPCR family C group 6 member A receptor (GPRC6A) [15,16,17]. Although the physiological function in improvement of glucose uptake and insulin sensitivity has been known, the interaction between the two cytokines remains unclear.

In this study, we used AdipoR1 transgenic mice as a model to demonstrate the role of ApN and Ocn in ovariectomized mice. Moreover, we also tested the expression pattern of GPRC6A in both pre- and post- menopausal mice to provide clues for the function of GPRC6A in menopause.

## Materials and methods

### Mouse menopausal-related metabolic disorder model

Generation and breeding strategies for AdipoR1 transgenic mice are described in a previous report [18]. The founder mice were crossed with wild-type (WT) mice to generate the F1 heterozygous (AdipoR1^+/−^) offspring and then backcrossed to the WT to generate the F2. When the F2 progeny were crossed, quantitative real-time PCR analyses was performed to identify the homozygous (AdipoR1^+/+^) littermates of the F3. All experiments were carried out on female homozygous offspring from the F4 or later generations. Mice, 6 weeks old were randomly housed in cages for each experimental group with the light-dark cycle maintained at 12:12h (lighting from 06:00 to 18:00 h). To generate a menopausal-related metabolic disorder model, 6-week-old female FVB/NJ mice and AdipoR1 transgenic mice were anesthetized by intraperitoneal injection of tribromoethanol (240 mg/kg) and the ovaries were removed by dorsal ovariectomy. After surgery, we continued to observe the situation of mice, if necessary, the analgesic treatment can be given (ibuprofen 30 mg/kg by oral administration). Body weight, glucose tolerance test, fasting glucose and serum Ocn (BT-479, Biomedical Technologies Inc., Stoughton, MA, USA) levels were measured 6 months after ovariectomy. Fasting glucose and glucose tolerance were measured after 16 hours fasting in ovariectomized wild-type and AdipoR1 transgenic mice. For sampling, the mice were anesthetized by tribromoethanol (intraperitoneally) and sacrificed by carbon dioxide and the peripheral tissues were be collected. All animal use and surgery protocols were approved by the institutional animal care and use committee at National Taiwan University.

### Western blot and immunofluorescence stain

Muscle tissue was homogenized in RIPA Lysis buffer (Millipore, Temecula, CA, USA). Twenty mg protein was used for sodium dodecyl sulfate polyacrylamide gel electrophoresis (90V, 120 min); separated proteins were then transferred onto a PVDF membrane (Perkin Elmer, Norwalk, CT, USA) at 400 mA for 90 min in transfer buffer (20% methanol, 192 mmol/L glycine and 25 mmol/L Tris/HCl). After transfer, the membrane was blocked with 1% bovine serum albumin for 2 h at room temperature. After three washes with phosphate buffered saline containing 0.1% Tween-20 (PBS/T), membranes were incubated with gentle agitation at 4°C overnight with the primary antibody at 1:1000 dilution for GPRC6A (Santa Cruz Biotechnology, Santa Cruz, CA, USA, sc-67302) or 1:5000 dilution for β-actin (Santa Cruz Biotechnology, Santa Cruz, CA, USA, sc-47778). After incubation with the primary antibody and three washes with PBS/T, the membrane was incubated with the appropriate secondary antibody at room temperature for 1 h. The membrane was then briefly incubated with a chemiluminescence reagent (ImmobilonWestern, Millipore, Billerica, MA, USA) and then exposed to the UVP BioSpectrum Imaging System (Upland, CA, USA). Target proteins were quantified by means of the UVP BioSpectrum Imaging System and referenced to the expression of β-actin in the same sample.

Immunofluorescence staining of 6 μm muscle tissue frozen sections used the primary antibody at 1:100 dilution for GPCR6A or AdipoR1 (Santa Cruz Biotechnology, Santa Cruz, CA, USA, sc-46748). After incubation with primary antibody overnight and three washes with PBS/T, the slides were incubated with the appropriate secondary antibody at room temperature for 1 h. After three washes with PBS/T, slides were mounted using UltraCruz® Mounting Medium with DAPI staining to detect nuclei (Santa Cruz Biotechnology, Santa Cruz, CA, USA, sc-24941).

### Micro-computed tomography (μCT) analysis

μCT analysis was as previously described [9]. Bone mineral density and parameters of trabecular bone were using the Skyscan 1076 scanner (BRUKER, Kontich, Belgium). The μCT measurements followed the guidelines for bone microstructure [19].

### Statistical analysis

All data were indicated as the mean ± SEM. Differences between two groups were analyzed by t-tests. Results involving more than two groups were analyzed by a one-way ANOVA procedure with the Dunnett Significant Difference test used to evaluate differences among means (GraphPad Prism 5, Version 5.01). A significant difference was considered at p ≤ 0.05.

## Results

### Overexpression of AdipoR1 improved insulin sensitivity in ovariectomized mice

**Fig 1:**
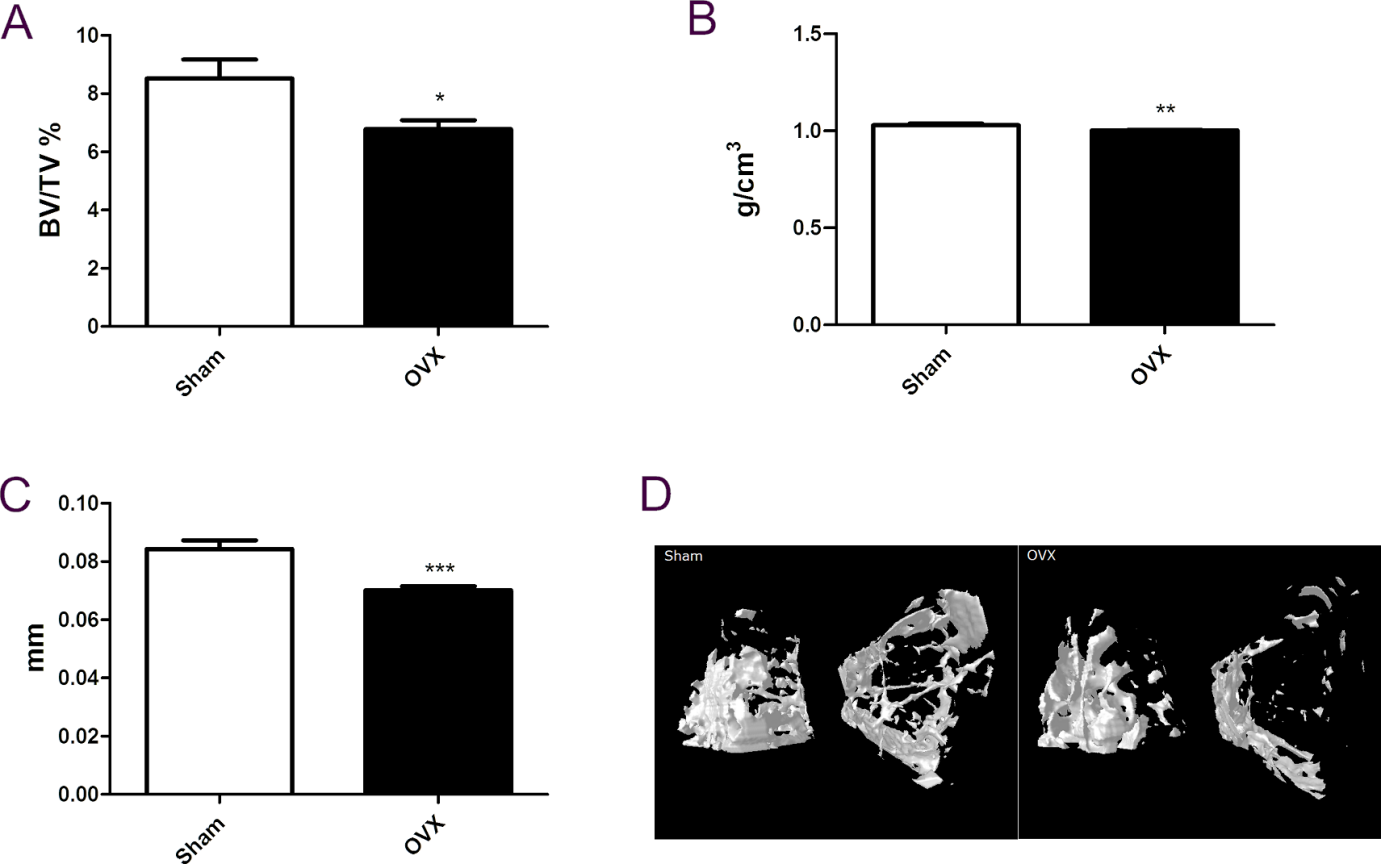
Model of postmenopausal metabolic disorder in mice. (A) Bone volume / trabecular volume %. (B) Bone density. (C) Trabecular thickness. (D) Representative metaphyseal microcomputed tomography images of mice of the indicated phenotype. OVX, ovariectomy. Data are expressed as mean± SEM. n = 6 to 8 Different letters indicate statistical significance. Symbols indicate statistical significance, *P ≦ 0.05, **P ≦ 0.01, and ***P ≦ 0.001 versus WT. The obtained “phantoms” with two known mass concentrations (0.25、0.75 g.cm-3) of CaHA (calcium hydroxyapatite). By linking these mass concentrations of CaHA with measured x-ray AC (attenuation coefficient) in the microCT image, and establish calibration linking the two quantities, allowing infer from the microCT measured attenuation coefficient in a mineralised tissue, the density of CaHA in g.cm-3. For bone volume, the 3d volume measurement is based on the hexahedral marching cubes volume model of the binaries objects within the VOI. And BV/TV (%) is the proportion of the VOI occupied by binaries solid objects.

**Fig 2:**
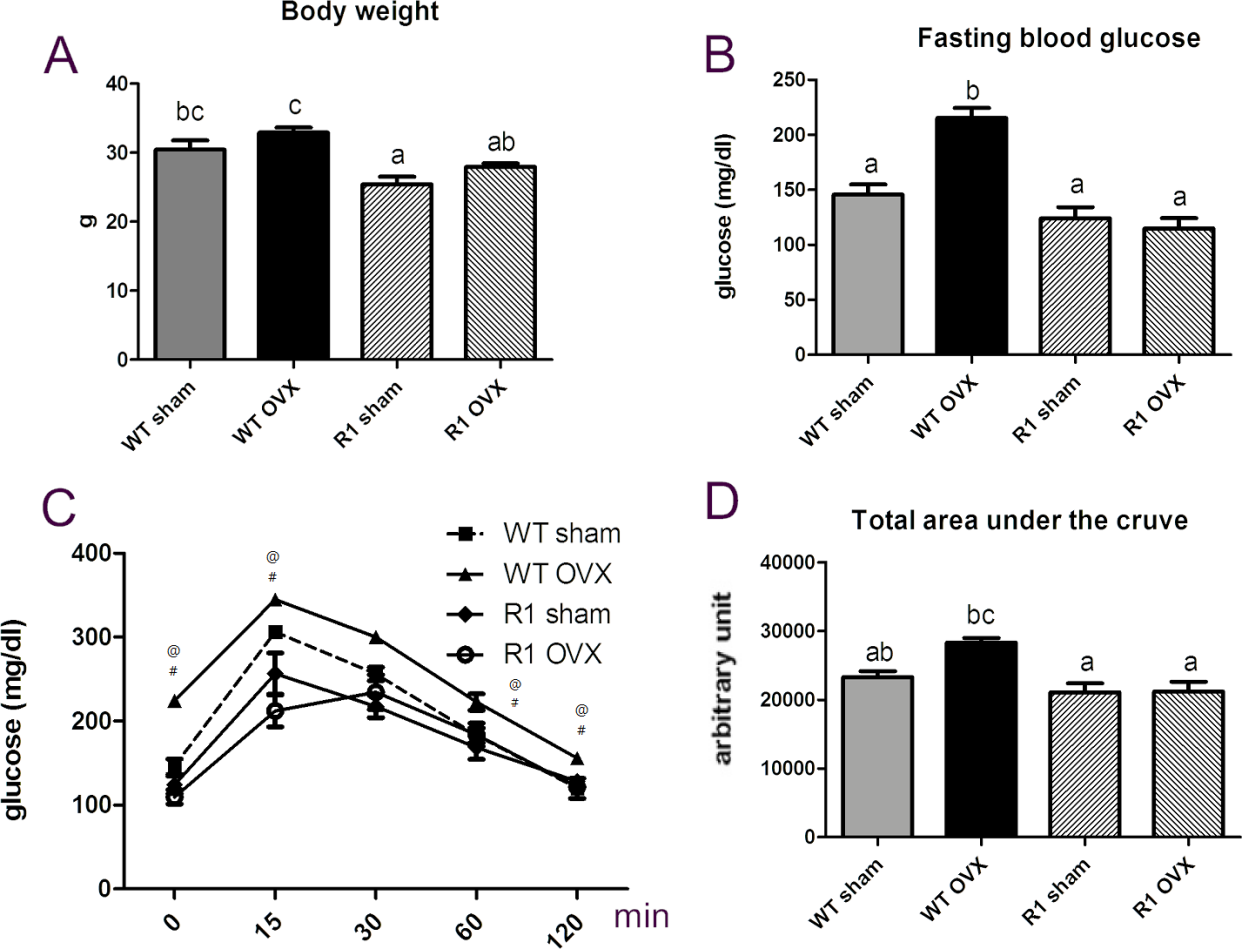
Overexpress AdipoR1 ameliorated glucose tolerance after ovariectomy. (A) Body weight. (B) Fasting blood glucose. (C) Glucose tolerance test. (D) Total area under the curve. WT, wild-type mouse; R1: adiponectin receptor 1 transgenic mouse; OVX, ovariectomy. Data are expressed as mean± SEM. n = 6 to 8 Different letters indicate statistical significance. p≦0.05:#, WT OVX vs. R1 OVX; @, WT OVX vs. WT sham.

**Fig 3:**
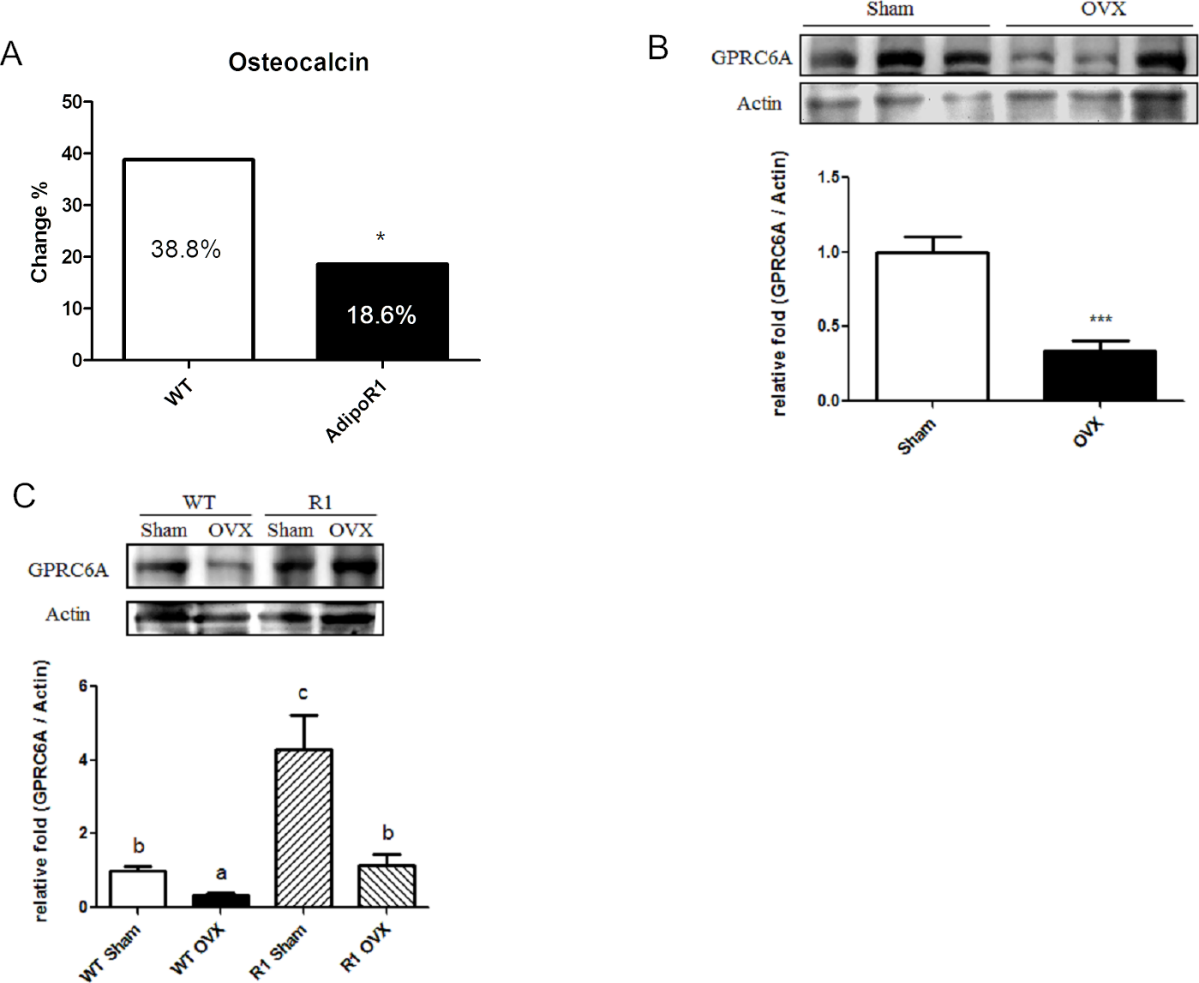
AdipoR1 transgenic ovariectomized mice have lower change of serum osteocalcin and higher GPRC6A expression than WT mice in muscle tissue. (A) Change of serum osteocalcin in WT and AdipoR1 transgenic mice after ovariectomy. (B) Expression of GPRC6A decreased after ovariectomy in mice. (C) AdipoR1 transgenic mice have higher GPRC6A expression after OVX. WT, wild-type mouse; R1: adiponectin receptor 1 transgenic mouse; OVX, ovariectomy. Data are expressed as mean ± SEM. n = 6 to 8 Different letters indicate statistical significance.

To explore the function of AdipoR1 in menopausal metabolic disorder, we used ovariectomized mice to mimic women at menopause. Fig 1A–1D indicated that ovariectomy produced decreased percent bone volume, bone mineral density and trabecular thickness. Overexpression of AdipoR1 decreased the fasting blood glucose level and improved insulin sensitivity in ovariectomized mice compared to WT mice (Fig 2B–2D). Overexpression of AdipoR1 also caused resistance to body weight increase (Fig 2A). Serum Ocn level was decreased after ovariectomy, whereas AdpoR1 transgenic mice had better ability to resist the decrease of serum Ocn than WT mice after ovariectomy (Fig 3A). We suggest that AdipoR1 may mediate the function of Ocn in glucose homeostasis in ovariectomized mice.

### AdipoR1 transgenic ovariectomized mice have higher GPRC6A expression in muscle tissue

We observed that GPRC6A protein expression was significantly decreased after ovariectomy (Fig 3B). Furthermore, AdipoR1 transgenic mice had greater muscle GPRC6A protein expression than WT mice at both pre- and post-ovariectomy stages (Fig 3C).

### The role between GPRC6A and AdipoR1 in muscle

**Fig 4:**
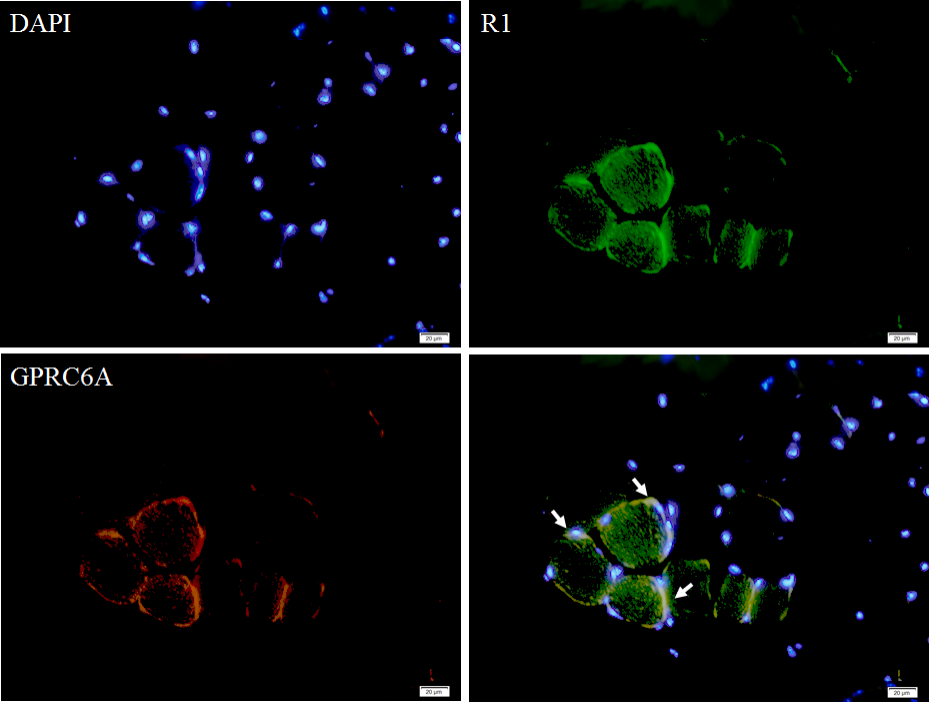
Immunofluorescence stain of GPRC6A and AdipoR1 in mouse muscle tissue. Cell nulei were stained with DAPI (blue). Muscle tissue sections were stained with GPRC6A (red) and AdipoR1 (green) antibody and viewed at X400 magnification with scale bars indicating 20 mm. Arrow refers to the co-localization of GPRC6A and AdipoR1.

Based on previous observations, we suggest that GPRC6A may play a role in mediating adiponectin signaling. We examined the distribution of GPRC6A and AdipoR1 in muscle tissue. Data from immunofluorescence showed that GPRC6A and AdipoR1 were co-localized in muscle tissue (Fig 4).

## Discussion

A function of Ocn is to improve glucose homeostasis by stimulation of β-cell proliferation and insulin secretion [7,12,13,14], but the insulin sensitized action in peripheral tissue has not been elucidated. In this study, in ovariectomized mice, we demonstrated that ApN signaling may mediate the function of Ocn in glucose homeostasis by interacting with GPRC6A, the putative receptor of Ocn. Our previous study showed that overexpression of AdipoR1 causes resistance to high fat / high sucrose diet induced-weight gain and glucose intolerance in mice [18]. Furthermore, AdipoR1 transgenic mice have higher serum Ocn than wild-type mice suggesting that Ocn may modulate glucose homeostasis via ApN signaling [9]. We observed that the decrease in serum Ocn in ovariectomized mice was less in AdipoR1 than in WT mice (Fig 3A). After ovariectomy, AdipoR1 transgenic mice had less body weight and fasting blood glucose, and improved glucose tolerance compared to WT mice (Fig 2). The results suggest that AdipoR1 mediated the Ocn function in glucose metabolism. In muscle tissue, in both pre- and post-ovariectomy mice, the protein expression of GPRC6A was significantly higher in AdipoR1 mice compared to WT mice (Fig 3B and 3C). The immunofluorescence data showed that AdipoR1 and GPRC6A were co-localized in muscle tissue (Fig 4) and phase contrast of muscle tissue showed in S1 Fig. Therefore, we speculated that GPRC6A had an interactive role with AdipoR1 or downstream adiponectin signaling which modulated glucose metabolism in the ovariectomized mice model.

Previous reports indicate that GPRC6A^-/-^ mice exhibit lower bone mineral density and higher susceptibility to develop the metabolic syndrome than WT mice [20,21]. Interestingly, there is no significant difference in serum Ocn and ApN in GPRC6A^-/-^ mice compared to WT mice suggesting that GPRC6A may exert its function downstream of ApN signaling to modulate glucose homeostasis [20,21]. Recently, studies *in vitro* show that expression of ApN is increased by Ocn treatment in adipocytes [22,23]. We examined the GPRC6A expression in adipose tissue in ovariectomized mice, and there has no difference between AdipoR1 transgenic mice and WT mice (data not shown). The role of GPRC6A in glucose homeostasis in adipocytes needs further investigation.

Our study, for the first time, suggests that AdipoR1 can mediate the function of Ocn on glucose metabolism in ovariectomized mice. Our findings suggest the possibility to ameliorate metabolic disorders through GPRC6A and ApN signaling.

## Supporting information

S1 Fig The phase contrast of immunofluorescence stain in mouse muscle tissue. (TIF)

S1 File NC3Rs ARRIVE guidelines checklist. (PDF)

S2 File Fig 1 dataset for all endpoint. (XLS)

S3 File Fig 2 dataset for all endpoint. (XLS)
