# Supplementary figures and images for: Adiponectin receptor 1 resists the decline of serum osteocalcin and GPRC6A expression in ovariectomized mice
Source: PLoS One. 2017 Dec 1;12(12):e0189063. doi: 10.1371/journal.pone.0189063 (PMC5711023; doi:10.1371/journal.pone.0189063)

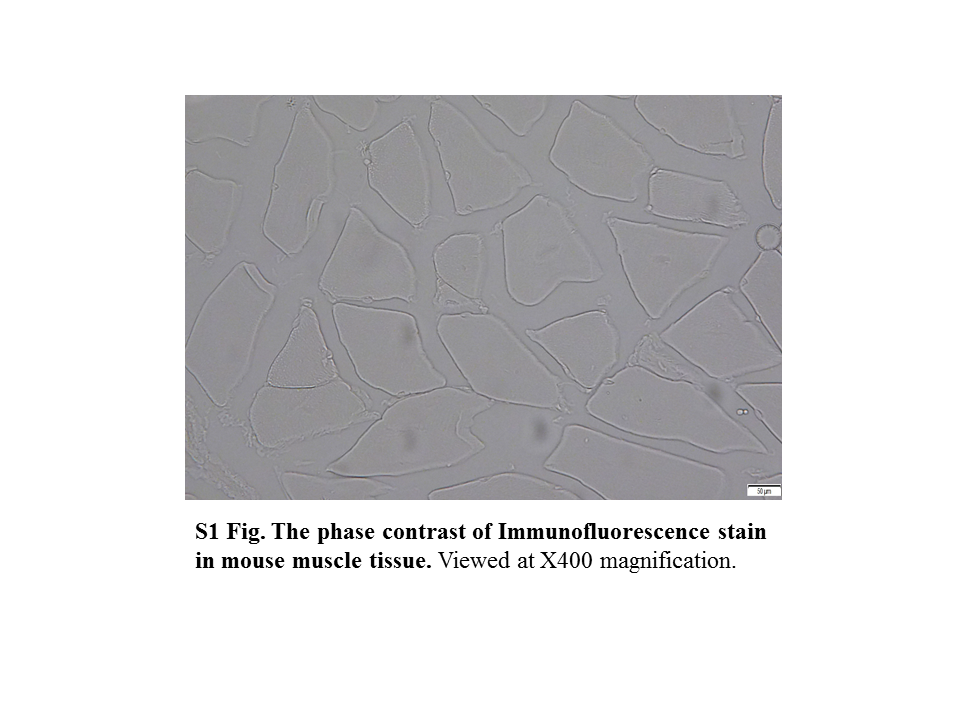

Supplement: S1 Fig — (TIF) [file pone.0189063.s001.tif]
